# Efficacy and safety of zanubrutinib plus R-CHOP in treatment of non-GCB DLBCL with extranodal involvement

**DOI:** 10.3389/fimmu.2023.1219167

**Published:** 2023-08-21

**Authors:** Hongzhi Geng, Sixun Jia, Ying Zhang, Jiaqi Li, Qin Yang, Liangyu Zeng, Xiangping Zong, Yutong Lu, Shuangzhu Lu, Jin Zhou, Caixia Li, Depei Wu

**Affiliations:** ^1^ National Clinical Research Center for Hematologic Diseases, Suzhou, China; ^2^ Jiangsu Institute of Hematology, First Affiliated Hospital of Soochow University, Suzhou, China; ^3^ Department of Hematology, Affiliated Zhongshan Hospital of Dalian University, Dalian, China; ^4^ Institute of Blood and Marrow Transplantation, Suzhou University Medical College, Suzhou, China; ^5^ Collaborative Innovation Center of Hematology, Soochow University, Suzhou, China

**Keywords:** zanubrutinib, untreated non-GCB DLBCL, extranodal involvement, anti-tumor activity, oncogenic mutations

## Abstract

**Introduction:**

Treatment with rituximab, cyclophosphamide, doxorubicin, vincristine, and prednisone (R-CHOP) shows poor response rates in non–germinal center B cell–like (non-GCB) diffuse large B-cell lymphoma (DLBCL) patients with multiple extranodal involvement. This study aims to evaluate anti-tumor activity and safety of zanubrutinib with R-CHOP (ZR-CHOP) in treatment naïve non-GCB DLBCL with extranodal involvement.

**Methods:**

In this single-arm, phase 2, prospective, single-center study, patients with newly diagnosed non-GCB DLBCL with extranodal involvement enrolled between October 2020 to March 2022 received ZR-CHOP for 6 cycles followed by 2 cycles of maintenance treatment with rituximab and zanubrutinib. The primary endpoint included progression-free survival (PFS) in the intent-to-treat (ITT) population whereas the secondary endpoints included overall response rate (ORR), complete response (CR), and duration of response. Further, next-generation sequencing (NGS) was used for detection of different oncogenic mutations closely related to DLBCL pathogenesis.

**Results:**

From October 2020 to March 2022, 26 patients were enrolled, and 23 of them were evaluated for efficacy after receiving 3 cycles of ZR-CHOP treatment. 1-year PFS and OS were 80.8% and 88.5% respectively while expected PFS and OS for 2-years are 74.0% and 88.5% respectively with median follow-up of 16.7 months and ORR was 91.3% (CR: 82.61%; PR: 8.70%). Oncogenic mutations closely related to DLBCL pathogenesis were assessed in 20 patients using NGS. B-cell receptor and NF-κB pathway gene mutations were detected in 10 patients, which occurred in MYD88 (7/19), CD79B (4/19), CARD11 (5/19), and TNFAIP3 (2/19). Hematological adverse events (AEs) ≥ grade 3 included neutropenia (50%), thrombocytopenia (23.1%), and anemia (7.7%) whereas non-hematological AEs ≥ grade 3 included pulmonary infection (19.2%).

**Conclusion:**

ZR-CHOP is safe and effective for treating treatment naïve non-GCB DLBCL patients with extranodal involvement.

**Clinical Trial Registration:**

Clinicaltrials.gov, NCT04835870

## 1 Introduction

Diffuse large B-cell lymphoma (DLBCL) is the most common type of non-Hodgkin lymphoma (NHL) and accounts for 30% to 40% of B-cell NHLs (B-NHL) (1). Gene expression profile has classified DLBCL into 3 molecular subtypes: germinal center B cell–like (GCB), activated B cell–like (ABC) and unclassifiable according to the cell of origin (COO) (2, 3). Alternatively, Hans algorithm is used to classify DLBCL into GCB and non-GCB subtypes based on approximation of immunohistochemistry (4). Studies have shown that the classification of DLBCL according to COO is an important prognostic factor in determining the treatment outcomes (5, 6). Non-GCB subtype showed significantly lower 5-year overall survival (OS) (54% vs. 78%) and progression-free survival (PFS) (48% vs. 76%) compared to GCB subtype (7).

Patients with DLBCL commonly present with a rapidly growing mass or enlarged lymph nodes at the nodal/extranodal site. Of all the cases of DLBCL, about one-third cases showed the primary extranodal sites of disease origin, whereas <70% showed involvement of at least one extranodal site. The most common extranodal sites of involvement include gastrointestinal tract, bones, testes, spleen, and central nervous system (CNS) (8–10). The presence of multiple extranodal involvements in DLBCL has been considered as an important factor in the International Prognostic Index (IPI) score because of associated poor prognosis (9).

In recent years, China has reported increased incidence of extranodal lymphomas, with non-GCB subtype DLBCL (70.7%) associated with significantly poor treatment response rates (75.5%) and overall survival rates (61.8%) as compared to single (84.6% and 80.4%) and no nodal involvement (89.3% and 81.3%) (11, 12). Rituximab in combination with CHOP regimen (R-CHOP) was used as a standard first-line treatment in DLBCL patients with a survival rate of 60% to 70%. However, 30% to 40% non-GCB DLBCL patients are either refractory or experience relapse in response to the first-line R-CHOP regimen and show poor prognosis and short OS rates (13–15). Therefore, newer treatment regimens with increased efficacy need to be developed for DLBCL patients non-responsive to routine first line treatment to achieve better remission and long-term survival.

There are several clinical trials to evaluate the effectiveness of new drugs alone or in combination with R-CHOP regimen in first-line treatment of DLBCL. However, currently only POLARIX study has shown PFS benefits with study drug compared to R-CHOP in first-line setting (16); in comparison with other studies which did not show any clear survival benefits. Nevertheless, different drugs were beneficial in different subgroups (Bcl-2 protein overexpression by immunohistochemistry, CD20+, ABC-type/non-GCB type DLBCL) in PFS, OS, or event-free survival (EFS) (17–19). Use of next-generation sequencing (NGS) in clinical studies to identify oncogenic mutations has enhanced our understanding about DLBCL heterogeneity which can facilitate precise diagnosis and effective treatment. In ABC subtype/non-GCB subtype of DLBCL, active B-cell receptor (BCR) signaling results in inhibition of apoptosis, thereby promoting uncontrolled cell division thus resulting in disease progression (PD), or can be responsible for insensitivity against chemotherapy (20–22). Non-GCB subtype tumors enriched for MCD (based on the co-occurrence of MYD88^L265P^ and CD79B mutations) and BN2 (based on BCL6 fusions and NOTCH2 mutations) subtypes can be treated using inhibitors targeting Bruton’s tyrosine kinase (BTK) present downstream to BCR and involved in activation of NF-κB activation (23, 24). In Phoenix study, the action of a first-in-class BTK inhibitor, ibrutinib, showed improved EFS and OS in in younger patients (aged <60 years) (19). According to the latest results, younger patients (aged <60 years) with BCR-dependent NF-κB pathway enriched MCD and N1 subtypes showed 100% 3-year EFS on treatment with ibrutinib plus R-CHOP (25). Thus, BTK inhibitors have shown promising results in the treatment of non-GCB DLBCL patients.

Zanubrutinib is a new type of small molecule oral BTK inhibitor, which can effectively inhibit BTK targets (26). In phase 1/2 clinical studies, zanubrutinib has shown promising results for safety and efficacy in patients with relapsed/refractory DLBCL (27). The present single-arm, phase 2, the prospective, single-center clinical trial has evaluated the efficacy and safety of the zanubrutinib in combination with R-CHOP in newly diagnosed non-GCB DLBCL with extranodal involvement (NCT04835870).

## 2 Materials and methods

### 2.1 Patients and treatment

Eligible patients are those aged >18 years with untreated non-GCB DLBCL with extranodal involvement, Eastern Cooperative Oncology Group performance status of 2 or lower, and main organ functions meeting the following conditions: left ventricular ejection fraction of ≥50%, creatinine clearance rate of ≥30 mL/min, levels of alanine transaminase (ALT) and aspartate transaminase (AST) ≤3 times the normal range, absolute neutrophil count of ≥1.0 × 10^9^/L cells, platelets count of ≥50 × 10^9^/L, and hemoglobin of ≥8.0 g/dL. Eligible patients should have expected survival time of ≥3 months

Key exclusion criteria included: Patients with (1) major surgery within 4 weeks pre-treatment; (2) primary mediastinal lymphoma or primary CNS lymphoma; (3) previous history of indolent lymphoma; (4) prior malignancy (other than DLBCL), except for cured malignant tumors with no active lesions for 3 years, and adequate treatment of inactive lesions in non-melanoma skin cancer, malignant tonsilloma, or carcinoma in situ; (5) history of intracranial hemorrhage in preceding 6 months and requiring or receiving anticoagulation with warfarin or equivalent antagonists; (6) Patients with underlying conditions that may increase their risk of receiving research drug treatment or confuse their judgment on toxic reactions as per researcher’s judgment.

### 2.2 Study design and treatments

This prospective, single-arm, single-center, phase 2 clinical trial was conducted on newly diagnosed non-GCB DLBCL with extranodal involvement to evaluate the efficacy and safety of the zanubrutinib combined with R-CHOP.

Enrolled patients received R-CHOP (intravenously rituximab [375 mg/m^2^ on Day 0], cyclophosphamide [750 mg/m^2^ on Day 1], doxorubicin [50 mg/m^2^ on Day 1], vincristine [1.4 mg/m^2^ on Day 1], and oral prednisone [50 mg/m^2^ on Days 1-5]) in combination with zanubrutinib (160 mg bid orally) in a cycle of 21 days for 6 cycles followed by 2 cycles of maintenance treatment including rituximab and oral zanubrutinib. Patients with a weak constitution or those aged >70 years were administered with ZR-miniCHOP treatment regimen. Patients with ≥3 score on Fatigue, Resistance, Ambulation, Illnesses, and Loss of Weight (FRAIL) scale were defined to have weak constitution. In total, 0.5 mg of entecavir was administered daily in occult carriers of hepatitis B virus (HBV) to prevent HBV reactivation.

The use of prophylactic granulocyte-colony stimulating factor (G-CSF) was not mandated. Therapeutic use of G-CSF 5 µg/kg daily was required until neutrophil count was close to near normal when grade ≥3 neutropenia was observed. Anti-infective medications to prevent recurrent viral, bacterial, or fungal infections were permitted but not mandated.

Pre-treatment clinical investigations included complete blood count, serum biochemistry with lactate dehydrogenase, HBV screening, HIV screening, and coagulation panel including activated partial thromboplastin time, prothrombin and fibrinogen time, bone marrow aspiration and trephine biopsy, electrocardiography, echocardiography, and positron emission tomography-computed tomography (PET-CT). GCB or non-GCB subgroups were determined using the Hans classification, with 30% cutoff values for CD10, BCL-6, and MUM-1. The cutoff value for BCL-2 was 50% and that for MYC was 40%.

Informed written consent was obtained from all patients. Study was conducted in compliance with the Declaration of Helsinki. The trial was overseen by the institutional steering and independent data monitoring committees. The study was approved by the ethics committee and institutional review board of the First Affiliated Hospital of Soochow University (Approval number: 2021035). This trial is registered with ClinicalTrials.gov, NCT04835870.

### 2.3 Endpoints and assessments

After 3 cycles of ZR-CHOP treatment, the therapeutic effect on each patient was evaluated by PET-CT response assessment. Interim and end of treatment PET-CT evaluations were performed using Deauville five-point score (5-PS) and percentage change of maximum standardized uptake value (ΔSUV_max_) predicting treatment outcome in DLBCL patients. Treatment was discontinued for patients with PD, recurrence, or who failed to achieve partial remission. The remaining patients continued treatment until the completion of 6 treatment cycles, PD, or intolerable toxicity.

The primary endpoint of the present study was PFS in the intent-to-treat (ITT) population based on Lugano 2014 criteria (28), defined as the time from enrollment to the first progression to death from any cause. The secondary endpoints included overall response rate (ORR), CR, and duration of response based on Lugano 2014 criteria, safety, and tolerability. In addition, the exploratory endpoints included use of NGS for identification of different oncogenic mutations. PET-CT was evaluated before treatment, interim of treatment, and at the end of induction for interim and final evaluation. Contrast-enhanced computed tomography (CT) of the neck, thorax, abdomen, and pelvis was repeated every 3 months thereafter to monitor PD until 1 year, then every 6 months until 2 years, and every year thereafter. Adverse events (AEs) were graded based on National Cancer Institute Common Terminology Criteria for Adverse Events (version 5.0).

### 2.4 Statistical analysis

The efficacy and the safety analyses were performed in the intention-to-treat (ITT) population. Nonparametric OS and PFS data of the ITT population were displayed using Kaplan-Meier curves. *P* value <0.05 indicated statistical significance. All analyses were performed using GraphPad Prism (version 9.0) and R software (version 4.0.1).

## 3 Results

### 3.1 Patient characteristics and treatment

**Figure 1:**
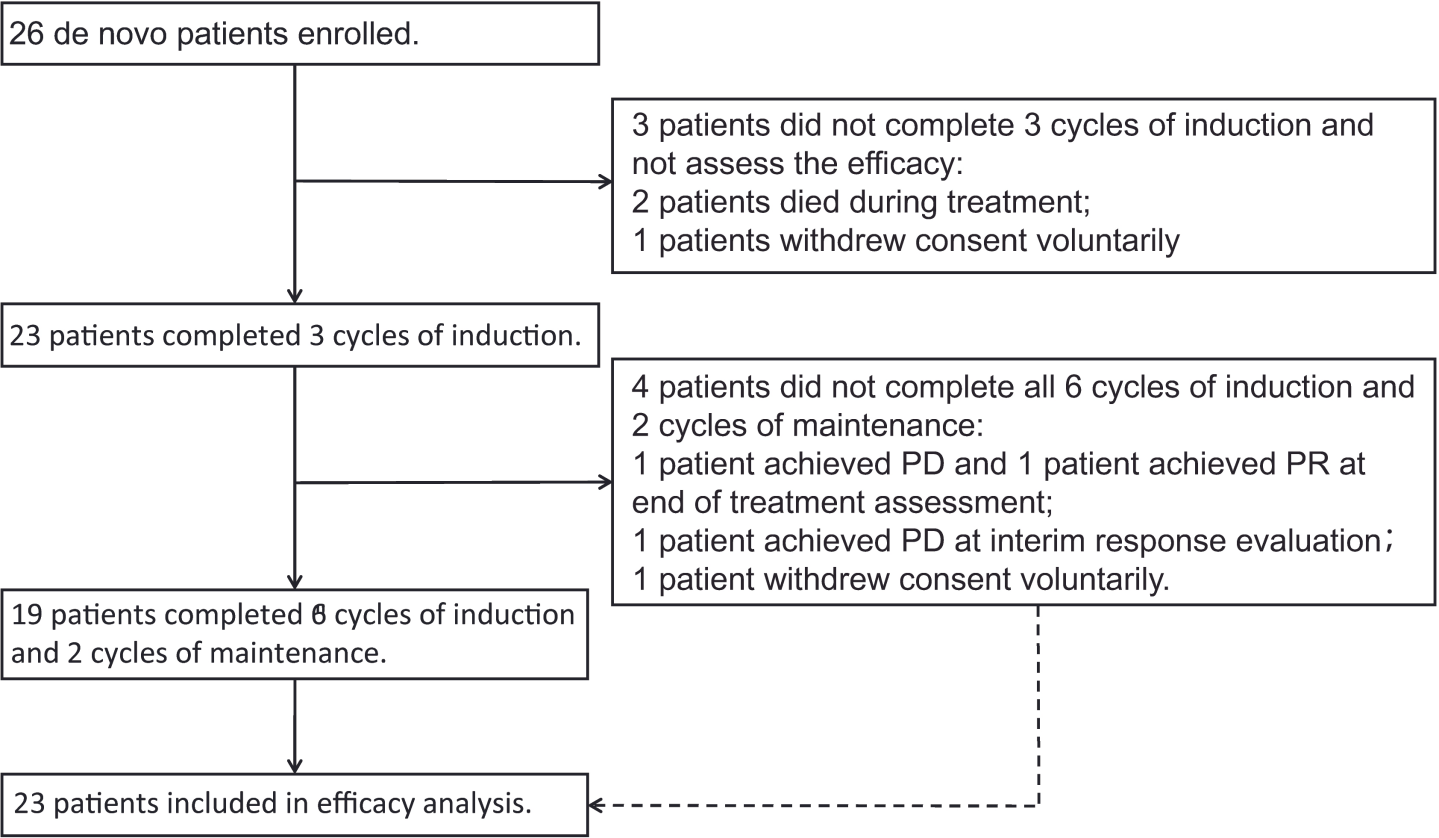
Patient flow diagram.

Between October 2020 and March 2022, 26 patients were enrolled in this study and received ZR-CHOP treatment. Detailed information regarding all the patients has been summarized in **Table 1**. All the patients were aged between 31 and 83 years, with a median age of 62 years. Of the total patients, 14 patients were aged >60 years. There were 14 patients with IPI- scores above 3. The incidence of stage III/IV disease was seen in 20 patients, and 15 patients showed involvement of ≥2 extranodal organs. There were 2 patients with bulky disease and 10 patients with double-expression lymphoma. (**Figure 1**). The median follow-up time for all patients was 16.7 months (1.7-28.2 months).

**Table 1A:** Detailed clinical information of enrolled patients.

	Sex	Age, years	Stage	IPI	Extranodal involvement	Other factors	Genetic subtype
1.	F	54	IIA	0	Colon	Double expression	Others
2.	M	52	IVB	3	Right thigh, lung, kidney	Bulky disease	ST2
3.	M	54	IVA	3	Spinal cord, testis	ND	BN2
4.	M	62	IVB	3	Bone marrow	ND	Others
5.	M	83	IIIA	3	Colon	Double expression	ND
6.	M	37	IVA	1	Skin nodule	ND	Others
7.	M	66	IVB	3	Colon, pleura	ND	BN2
8.	M	51	IA	0	Testis	ND	ND
9.	M	53	IIA	0	Colon	ND	ST2/N1 composite
10.	F	68	IVA	4	Peritoneum, ileum	Bulky disease	MCD
11.	M	31	IIA	0	Pharynx	ND	ND
12.	M	71	IVA	2	Testis	Double expression	MCD/BN2 composite
13.	M	62	IVB	4	Seminal vesicle, rectum, prostate, bladder, ureter	ND	BN2
14.	M	69	IVA	3	Skin nodule, parotid	Double expression	MCD
15.	M	56	IVA	4	Colon, peritoneum	ND	N1
16.	M	63	IVA	1	Lung, pleura, adrenal	ND	ND
17.	M	53	IVB	3	Nasopharynx, epiglottis, adrenal	Double expression	MCD
18.	F	77	IVA	3	Right thigh, skin nodule	Double expression	MCD
19.	M	58	IIIA	1	Ileum	Double expression	ND
20.	F	73	IIA	3	Nasopharynx, sinus	Double expression	Others
21.	F	64	IIA	1	Breast	ND	Others
22.	F	63	IVA	3	Ileum, adrenal gland	ND	Others
23.	F	55	IVA	2	Bone marrow, bone	ND	Others
24.	M	78	IVA	3	Nasopharynx, testis	ND	ND
25.	F	64	IVA	5	Pancreas, peritoneum, bone marrow	Double expression	Others
26.	F	37	IIA	1	Thyroid	ND	ST2

BN2, BCL6 translocations and NOTCH2 mutations; IPI, International Prognostic Index; MCD, MYD88^L265P^ and CD79B mutations; N1, NOTCH1 mutations; ND, not determined; ST2, SGK1 and TET2 mutations. DLBCL staging according to Lugano classification: Stage I (involving one lymph node region or extra lymphatic site); Stage II (involving two or more lymph nodes on the same side of the diaphragm); Stage III (involving lymph nodes on both the sides of the diaphragm); Stage IV (spreads into one or more extra-lymphatic organs like bone marrow, liver, lung with or without the involvement of the lymph node.).

**Table 1B:** Consolidated information baseline characteristics of enrolled patients.

Characteristics	N=26
Cell of Origin (Hans)	
GCB	0
NonGCB	26
Gender	
Male	17
Female	9
Age	
Median(range)	62(31~83)
<60	12
≥60	14
Ann Arbor stage	
I/II	6
III/IV	20
Number of extranodal involvement	
1	9
≥2	17
IPI	
0~2	11
3~5	15
Double Expression	
Yes	9
No	17
ECOG	
0~1	
2	
Sum	26

### 3.2 Efficacy

**Figure 2:**
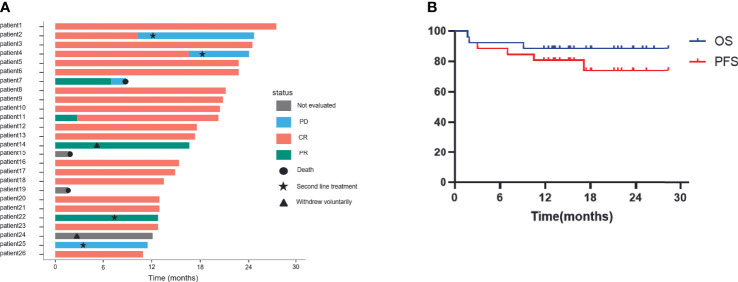
Efficacy of patients treated with ZR-CHOP depicting **(A)** disease progression and **(B)** OS and PFS of all patients. CR, complete response; PR, partial response; PD, progression disease; ZR-CHOP: Zanubrutinib combined with rituximab, cyclophosphamide, doxorubicin, vincristine, and prednisone. Includes efficacy outcomes for 23 patients.

Overall, 23/26 patients could be evaluated for efficacy. The remaining patients withdrew from the clinical trial or died before reaching the primary efficacy endpoint - 2 patients died of causes other than disease progression (one patient died suddenly and another one died of familial hereditary cerebral hemorrhage) before efficacy evaluation and 1 patient withdrew from the treatment voluntarily after 2 cycles of the treatment. Out of 23 patients assessed, 21 patients showed an objective response rate (ORR) of 91.3%. Complete remission was seen in 19 patients (82.6%), partial remission in 2 patients (8.7%), and PD in 2 patients (8.7%), 1-year PFS and OS were 80.8%, 88.5% respectively and expected PFS and OS for 2-years are 74.0% and 88.5% respectively with a median follow-up of 16.7 months and the median DOR as of the last follow-up was 13.5 months(3.2~25.1months). The specific situation of each patient and Kaplan-Meier plots depicting survival outcomes are shown in **Figure 2**. Among the 2 patients who died during the treatment, only one patient experienced progression of the CNS after treatment and died within a short period of time.

**Figure 3:**
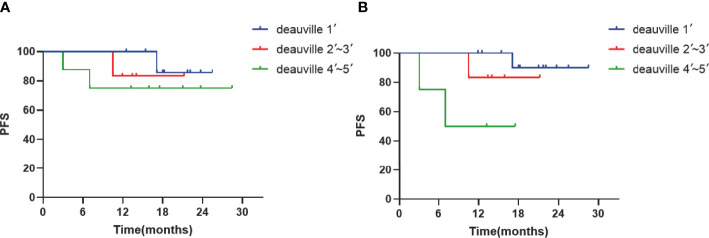
PFS according to response at **(A)** interim PET-CT and **(B)** end of treatment PET-CT.

All patients were assessed for interim and end of treatment efficacy using PETCT. The median ΔSUV_max_ value of interim and end-of-treatment were 91.2% (67.6%-100%) and 100% (84.3%-100%), respectively (**Table 2**), performed at the end of the treatment in patients responding to ZR-CHOP treatment regimen with at least PR. There were 56.5% of patients (n = 13) who showed CR with a Deauville score of 1. Statistical analysis was conducted on patients with different responses, and the results showed that patients who achieved CR with a Deauville score of 1 were more likely to achieve more lasting response (**Figure 3**).

**Table 2:** Results of interim and final PET-CT response evaluation.

	Sex	Age (years)	Before treatment		Interim evaluation			End of treatment		
			Surgery	SUV_max_	Efficacy	ΔSUV_max_	5-PS	Efficacy	ΔSUV_max_	5-PS
1.	F	54	Yes	18.1	PR	67.6%	4	CR	100%	1
2.	M	52	No	20.1	CR	90.2%	3	CR	90.2%	3
3.	M	54	Yes	–	CR	100%	1	CR	100%	1
4.	M	62	No	11.0	CR	100%	1	CR	100%	1
5.	M	83	No	13.7	PR	65.7%	5	CR	100%	1
6.	M	37	No	26.4	CR	100%	1	CR	100%	1
7.	M	66	No	16.4	PR	86.6%	4	PD	ND	ND
8.	M	51	Yes	–	CR	100%	1	CR	100%	1
9.	M	53	No	15.3	CR	100%	1	CR	100%	1
10.	F	68	Yes	22.3	CR	91.4%	3	CR	94.1%	3
11.	M	31	No	18.4	PR	78.3%	4	CR	100%	1
12.	M	71	Yes	15.6	CR	100%	1	CR	100%	1
13.	M	62	No	25.2	CR	100%	1	CR	100%	1
14.	M	69	Yes	22.7	PR	79.7%	5	ND	ND	ND
15.	M	56	ND	ND	ND	ND	ND	ND	ND	ND
16.	M	63	No	14.1	PR	79.4%	4	CR	90.8%	3
17.	M	53	No	13.8	CR	100%	1	CR	100%	1
18.	F	77	No	8.3	CR	79.5%	3	CR	84.3%	3
19.	M	58	ND	ND	ND	ND	ND	ND	ND	ND
20.	F	73	No	20.5	CR	90.7%	3	ND	91.6%	3
21.	F	64	No	16.2	CR	94.4%	2	CR	91.4%	3
22.	F	63	Yes	29.9	PR	91.0%	4	ND	93.3%	4
23.	F	55	Yes	ND	CR	100%	1	CR	100%	1
24.	M	78	ND	ND	ND	ND	ND	ND	ND	ND
25.	F	64	No	13.4	PD	ND	ND	ND	ND	ND
26.	F	37	No	7.8	CR	75.6%	3	CR	100%	1

CR, complete response; ND, not determined; F, female; M, male; PET-CT, positron emission tomography-computed tomography; PR, partial response; PD, progression disease; SUV_max_, maximum standardized uptake values; ΔSUV_max_, change in SUV_max_ values before and after treatment; 5-PS, five-point scale Deauville score.

### 3.3 Identification of onogenic mutations in DLBCL patients using next-generation sequencing (NGS)

**Figure 4:**
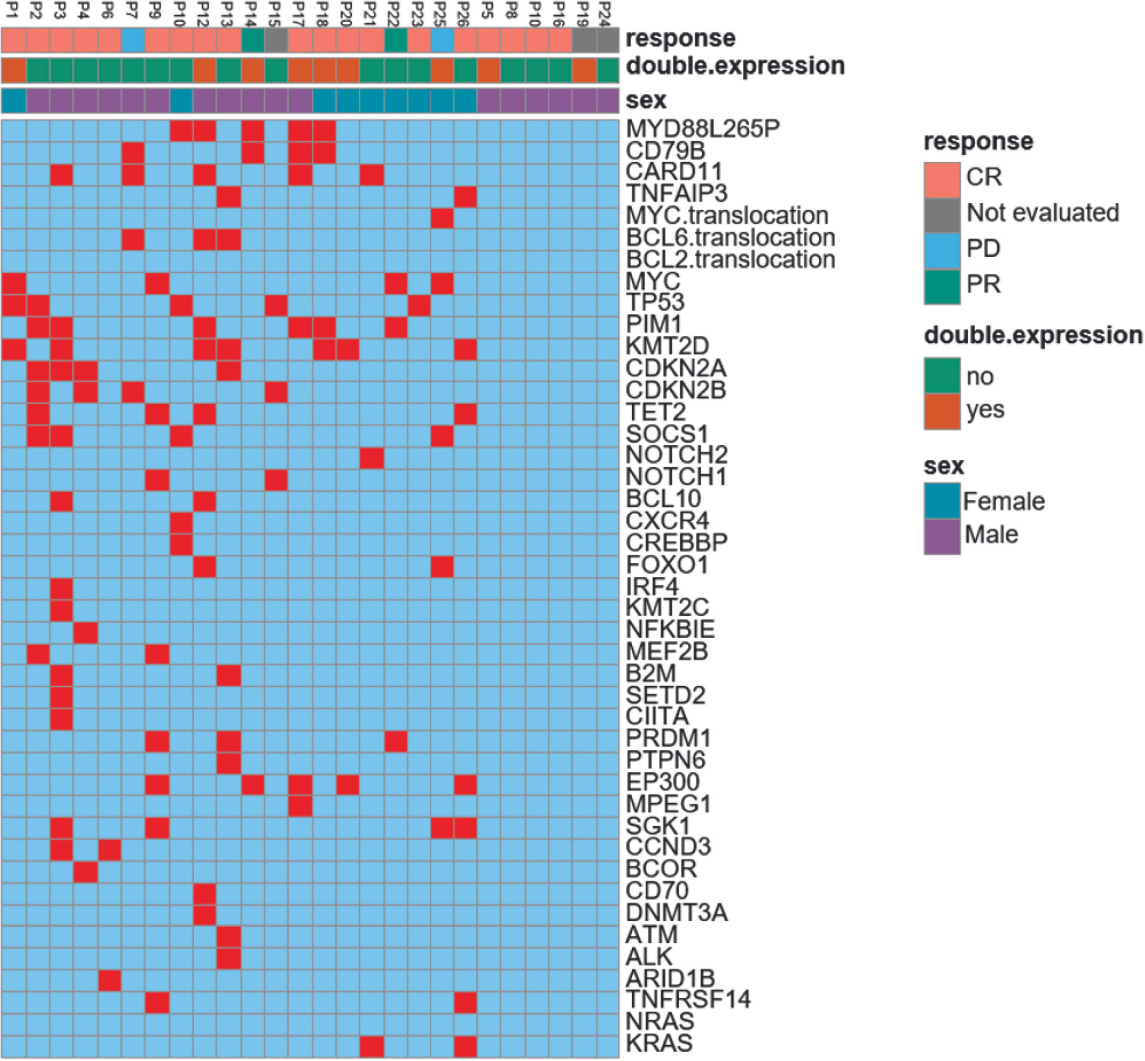
Genetic lesions and response of patients. CR, complete response; PR, partial response; PD, progression disease. Includes analysis of genetic lesions and response for 23 patients.

Of 26 patients, 20 (76.9%) were assessed for oncogenic mutations closely related to DLBCL pathogenesis. The genetic lesions and related efficacy for each patient are presented in **Figure 4**. According to LymphGen, there were 4 cases of MCD, 3 cases of BN2, 2 cases of ST2, 1 case of N1, 1 case each of MCD/BN2 and sT2/N1 composite, and 8 cases of others among assessed patients as per their genotype profile. BCR and NF-κB pathway gene mutations were detected in 10 patients. Mutations in MYD88 were seen in 7 patients (35%), CD79B in 4 patients (20%), CARD11 in 5 patients (25%), and TNFAIP3 in 2 patients (10%). MYD88 mutations were detected in 7 patients, of them, 5 patients showed MYD88L265P mutations and 3 patients showed CD79B double mutations. There were IRF4 and NOTCH1 mutations in 1 patient each and ATM mutations in 2 patients from the phosphatidylinositol 3 kinase-AKT pathway. Mutations in the cell cycle or p53 pathway were observed in CCND and FAS in 2 patients each, TP53 in 5 patients, MYC in 4 patients, and NFKBIE in 1 patient. Histone or DNA methylation gene mutations occurred in TET2 in 3 patients and KMT2D in 7 patients, whereas histone acetylation gene mutations occurred in CREBBP in 1 patient and EP300 in 5 patients. Chromatin remodeling gene mutations occurred in SGK1 in 3 patients. T-cell activation gene mutations occurred in PRDM1 in 3 patients and TNFRSF14 in 2 patients. IFN-γ response pathway gene mutations occurred in SOCS1 in 4 patients as well as B2M and CIITA in 1 patient each. Survival analysis was not performed due to the limited sample size.

### 3.4 Safety

Out of total 26 patients evaluated for safety outcomes, dose reduction of Zanubrutinib was seen in 14 patients (53.8%). AEs were the most common reason for dose reduction or treatment discontinuation. AEs classified based on their severity are enlisted in **Table 3**. Hematological AEs with severity ≥ grade 3 reported were neutropenia (13 patients, 50%), thrombocytopenia (6 patients, 23.1%), and anemia (2 patients, 7.7%), 4 patients (15.9%) observed febrile neutropenia. Pulmonary infection (5 patients, 19.2%) was the ≥ grade 3 non-hematological AE observed in this study. No patient presented with severe liver and kidney dysfunction. Bleeding (3 patients, 11.5%) and cardiovascular events (2 patients, 7.7%) were observed upon oral administration of zanubrutinib. Out of 3 patients who suffered with hemorrhage, none had severe or even fatal hemorrhage. Patients with atrial fibrillation recovered their sinus rhythm after cardiovascular treatment and were further continued on orally administered zanubrutinib. No cardiovascular and cerebrovascular adverse reactions were observed in other patients. No patients were with fatal AEs. Besides, all patients received necessary antibiotic prophylaxis to manage the incidence of AEs.

**Table 3:** Adverse events observed in all patients.

Adverse events (N=26)	ZR-CHOP treatment regimen, n (%)		
	All grade	Grade 1/2	Grade 3/4
Hematological toxicities			
Neutropenia	18 (69.2%)	5 (19.2%)	13 (50.0%)
Febrile neutropenia	4 (15.9%)	0	4 (15.9%)
Anemia	18 (69.2%)	16 (61.5%)	2 (7.7%)
Thrombocytopenia	15 (57.7%)	9 (34.6%)	6 (23.1%)
Non-hematological toxicities			
Pulmonary infection	6 (23.1%)	1 (3.8%)	5 (19.2%)
Atrial fibrillation	2 (7.7%)	1 (3.8%)	1 (3.8%)
Hyperuricemia	5 (19.2%)	5 (19.2%)	0
ALT or AST elevation	3 (11.5%)	3 (11.5%)	0
Elevated creatinine	1 (3.8%)	1 (3.8%)	0
Hemorrhage	3 (11.5%)	3 (11.5%)	0
Nausea	9 (34.6%)	9 (34.6%)	0
Apositia	12 (46.1%)	12 (46.1%)	0
Fatigue	14 (53.8%)	14 (53.8%)	0
Diarrhea	3 (11.5%)	3 (11.5%)	0

ZR-CHOP: Zanubrutinib combined with rituximab, cyclophosphamide, doxorubicin, vincristine, and prednisone; N: number of patients assessed.

## 4 Discussion

The present study reported a single-arm, prospective, single-center, phase 2 trial for newly diagnosed non-GCB DLBCL with extranodal involvement to evaluate the efficacy and safety of the ZR-CHOP treatment regimen. We have observed satisfactory therapeutic effects in patients of different ages, although without a large sample. This study showed an ORR of 91.3% (21/23), of which, CR was 82.61%, 1-year PFS and OS were 80.8% and 88.5% respectively while expected PFS and OS for 2-years are 74.0% and 88.5% respectively in ITT population with median follow-up of 16.7 months and the median DOR as of the last follow-up was 13.5 months (3.2~25.1months). Although the overall sample size may not be enough, we still observe a good benefit trend in these patients. The proportion of patients with grade ≥3 neutropenia in the R-CHOP cohort of GOYA study was 39.5%. While the present study observed a higher proportion of patients experiencing neutropenia (50%) than GOYA study, the proportion remained roughly the same for febrile neutropenia in both the studies (15.9% vs. 15.4%) (29). In the Phoenix study, more serious AEs (SAEs) were reported in the ibrutinib plus R-CHOP than in the placebo plus R-CHOP arm (19). Although almost no grade ≥3 neutropenia was observed in zanubrutinib monotherapy reported by Yang et al. (27), the combination of medications might have led to additional hematological toxicities. It has been reported that primary prophylaxis with pegfilgrastim tended to have a lower odds ratio for the occurrence of febrile neutropenia in patients aged 50-70 years and was significantly associated with reduced febrile neutropenia occurrence in patients aged 70-80 years (30). Therefore, the slight increase in the incidence of hematological toxicities might also be due to no use of prophylactic G-CSF. Crucially, these additional events were manageable using opportune symptomatic treatment such as anti-infection and G-CSF and did not translate into an increased risk of fatal AEs and death.

Non-GCB subtype of DLBCL with high Ki-67 index and overexpression of MYC and BCL2 proteins (double expression), insensitive to the standard first-line treatment show poor prognostic significance (31). Also, previous studies have shown positive correlation between high MYC and concurrent high MYC/BCL2 double-expression with individual markers of active BCR signaling, thereby showing enhanced BCR activation (32, 33). Similar results were seen in the present study. BCR and NF-κB pathway gene mutations were detected in 4 of the 8 patients with double-expression lymphoma, which showed effective response on zanubrutinib treatment. Form these 8 double-expression lymphoma patients, there were 7 patients with effective post-treatment response, 6 patients achieved CR, whereas all of them obtained continuous remission. Therefore, the use of BTK inhibitor has effectively helped in controlling increased BCR activation in these patients with BCR and NF-κB pathway gene mutations and thereby achieved satisfactory results. No incidence of increased risk of severe infection and hemorrhage was reported in patients on zanubrutinib regimen in combination with R-CHOP. Moreover, no serious AEs and fatal events were observed as well. The presence of zanubrutinib-related atrial fibrillation and skin and mucosal hemorrhage was manageable and did not lead to dose reduction or treatment discontinuation.

The BCR signaling pathway is widespread and plays an exceedingly vital role in the development of DLBCL, especially the ABC subtype of DLBCL relies on chronic active BCR signaling and the survival of ABC DLBCL cells depends on the BCR signaling pathway (21, 22). As zanubrutinib is a BTK inhibitor directly acting on BCR signaling, this treatment has been effective in a majority of patients. Previous studies have shown BCR-dependent NF-κB activation to be common in MCD and BN2 tumors. The frequency of MYD88 and CD79b mutations has been observed to be high in patients with primary extranodal involvement. All of these patients have shown a poor prognosis in response to the standard immunotherapy (12). To test the genetic predisposition of patients to treatment efficacy, oncogenic mutations closely related to DLBCL pathogenesis were assessed in 20 (76.9%) of 26 patients. TP53 mutations were detected in 5 patients. There was total 10 patients with BCR and NF-κB pathway gene mutations, involving MYD88, CD79B, CARD11, and TNFAIP3. Among these patients, 8 of 10 achieved a sustained response. Therefore, patients with these genetic lesions were effectively treated with BTK inhibitors and could achieve a better clinical effect. For other DLBCL patients with PD or relapse, their genetic mutations were involved in histone or DNA methylation, cell cycle, or p53 and not in BCR or NK-κB pathway. Since these mutations are nowhere linked with BCR signaling, BTK inhibitors may not be the best choice to effectively treat these patients.

Treatment responses have been analyzed by interim and end of treatment PET-CT response evaluation using Deauville scores. While the evaluation of interim PET-CT analysis using Deauville score has been observed to be useful in determining treatment prognosis in several clinical and retrospective studies (34, 35), there are studies reporting contradictory results as well (36–38). Kurch et al. have previously reported that lower Deauville score for the end of treatment PET-CT evaluation is associated with superior treatment response in patients with similar prognosis (37). In addition, ΔSUV_max_ is another parameter commonly used to evaluate treatment response with different implications involved in prediction of patient recurrence or survival at different thresholds (35, 36, 39, 40). Although the results from the present study do not show any unified conclusion, the trend observed in the results denotes that high ΔSUV_max_ could be a measure for better PFS and OS. The present study showed posttreatment median ΔSUV_max_ of 91.2% (67.6%-100%), which was higher than the median ΔSUV_max_ of 66% (41), 70% (41), even 82% (35) reported in the previous studies. This denotes relatively satisfactory (ideal) remission in patients after treatment. However, these results are not statistically significant, which may be due to lack of patients and follow-up time. This study hopes to get more predictive values from the medium-term PET-CT to make prompt adjustments in the treatment of patients with poor prognoses. Follow-up of these patients will be continued along with performing multicenter study for further verification.

In conclusion, this study demonstrated an encouraging clinical benefit and a tolerable safety of ZR-CHOP regimen in newly diagnosed DLBCL patients with extranodal involvement. Furthermore, this study provides evidence that ZR-CHOP regimen could enable more high-risk patients to achieve better efficacy at the end of the first-line treatment and may make it possible for some patients to get long-time remission in the first-line treatment. However, these results will need further confirmation through a large sample size randomized clinical trial.

## Data availability statement

The original contributions presented in the study are publicly available. This data can be found here: National Genomics Data Centre (NGDC), GSA, accession number HRA005297. Further inquires can be directed to the corresponding author/s.

## Ethics statement

The studies involving human participants were reviewed and approved by ethics committee and institutional review board of the first affiliated hospital of Soochow University (Approval number: 2021035). The patients/participants provided their written informed consent to participate in this study.

## Author contributions

YZ, JL, CL, and DW contributed to conception and design. XZ, YL, SL, JZ, and DW provided study material or patients. All authors participated in the collection and assembly of data. All authors analyzed and interpreted the data. HG wrote the manuscript. All authors gave final approval of the manuscript and agreed to be accountable for all aspects of the work.
